# B Cell Subsets in Atherosclerosis

**DOI:** 10.3389/fimmu.2012.00373

**Published:** 2012-12-11

**Authors:** Heather M. Perry, Timothy P. Bender, Coleen A. McNamara

**Affiliations:** ^1^Department of Pathology, University of VirginiaCharlottesville, VA, USA; ^2^Cardiovascular Research Center, University of Virginia Health SystemCharlottesville, VA, USA; ^3^Department of Microbiology, Immunology and Cancer Biology, University of VirginiaCharlottesville, VA, USA; ^4^Beirne B. Carter Center for Immunology ResearchCharlottesville, VA, USA; ^5^Cardiovascular Division, Department of Internal Medicine, University of Virginia Health SystemCharlottesville, VA, USA

**Keywords:** B cell, atherosclerosis, IgM, cytokines, lipids

## Abstract

Atherosclerosis, the underlying cause of heart attacks and strokes, is a chronic inflammatory disease of the artery wall. Immune cells, including lymphocytes modulate atherosclerotic lesion development through interconnected mechanisms. Elegant studies over the past decades have begun to unravel a role for B cells in atherosclerosis. Recent findings provide evidence that B cell effects on atherosclerosis may be subset-dependent. B-1a B cells have been reported to protect from atherosclerosis by secretion of natural IgM antibodies. Conventional B-2 B cells can promote atherosclerosis through less clearly defined mechanism that may involve CD4 T cells. Yet, there may be other populations of B cells within these subsets with different phenotypes altering their impact on atherosclerosis. Additionally, the role of B cell subsets in atherosclerosis may depend on their environmental niche and/or the stage of atherogenesis. This review will highlight key findings in the evolving field of B cells and atherosclerosis and touch on the potential and importance of translating these findings to human disease.

## Introduction

**Figure 1:**
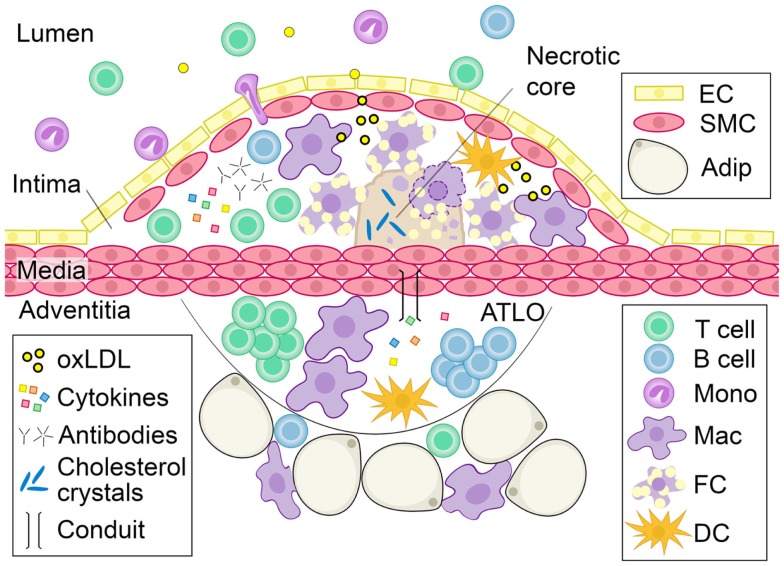
**Characteristics of advanced atherosclerotic disease**. As luminal LDL is deposited into the subendothelial space of the blood vessel wall, it becomes oxidized. Oxidized lipids trigger the recruitment of leukocytes to the subendothelial space. Monocytes differentiate into dendritic cells or tissue macrophages that take up oxidized lipids and become foam cells. Without effective clearance of these apoptotic-prone cells, they accumulate, secrete pro-inflammatory cytokines and undergo apoptosis and necrosis, dumping their lipid contents into the extracellular space to create a necrotic core. Cytokines and immunoglobulins produced by vessel wall and immune cells can further modulate atherosclerosis. ATLOs are found in the adventitia of diseased vessels and are composed of T cells, B cells, macrophages, and other leukocytes. Soluble factors can cross into the media through conduits and may contribute to plaque development. Abbreviation: oxLDL, oxidized LDL; ATLO, aortic tertiary lymphoid organ; EC, endothelial cell; SMC, smooth muscle cell; Adip, adipocyte; Mono, monocyte; Mac, macrophage; FC, foam cell; DC, dendritic cell.

Atherosclerosis and its attendant sequelae of heart attacks and strokes remains a leading cause of death and disability in Westernized countries (Roger et al., 2012). Substantial work over the last several decades has clearly established atherosclerosis as a chronic inflammatory disease of the blood vessel wall (Figure 1). Immune cells including macrophages, dendritic cells, mast cells, neutrophils, T cells, and B cells regulate the atherogenic process (Libby, 2002, 2012; Hansson and Hermansson, 2011; Lahoute et al., 2011). As such, immunomodulatory therapy holds promise as the next frontier for improving prevention of atherosclerotic cardiovascular disease (Keaney, 2011; Libby et al., 2011; Weber and Noels, 2011). Deposition of lipids such as low-density lipoprotein (LDL) in the subendothelial space of the intima and other injurious stimuli have been implicated as initial inflammatory triggers (Lusis, 2000). Vessel wall enzymes can act on deposited lipids to generate modifications in the lipids, such as oxidation, that serve both directly and indirectly as inflammatory signals (Hansson et al., 2006; Chou et al., 2008; Steinberg and Witztum, 2010; Miller et al., 2011). Inflammatory cells are then recruited to the arterial wall, promoting progression of plaques through a host of interconnected mechanisms (Weber et al., 2008; Galkina and Ley, 2009; Tabas, 2010; Hansson and Hermansson, 2011; Murray and Wynn, 2011). For example, recruited monocytes differentiate into macrophages within the plaque. There they engulf oxidized lipids, becoming foam cells, which secrete chemokines and other cytokines that further promote immune cell infiltration and activation. In addition, many of these lipid-laden macrophages subsequently undergo apoptosis and necrosis, dumping their contents into the extracellular space, creating a necrotic core (Figure 1). Formation of the necrotic core promotes plaque expansion and, if not contained, can lead to the unstable syndromes that result in heart attack and stroke. In addition to immune cell entry into the plaque via the blood vessel lumen, immune cells are also found in the adventitia, or outer layer of the vessel wall. In fact, aortic tertiary lymphoid organs (ATLOs) have been identified in the aortic adventitia of aged mice at sites of advanced intimal plaque. Conduit networks, similar to those that filter soluble substances within lymph nodes (Sixt et al., 2005) and facilitate lymphocyte organization in the white pulp of the spleen (Nolte et al., 2003) connect the adventitia with the vessel wall (Figure 1). The role of the adventitia in regulating atherosclerosis has been reviewed elsewhere (Campbell et al., 2012; Weih et al., 2012).

Lymphocytes have long been identified in the adventitia and plaque of diseased arteries. In his 1915 book, *Diseases of the Arteries, Including Angina Pectoris*, Sir Thomas Clifford Allbutt noted that, “Round cell growth in the adventitia in arteriosclerosis is correlated with absorption of depraved matter from the diseased intima” (Allbutt, 1915), suggesting that lymphocytes might serve to protect against the accumulation of lipids and necrotic material in blood vessel walls. Subsequent histological studies confirmed the presence of lymphocytes in diseased blood vessels, underscoring the hypothesis that lymphocytes may be important regulators of atheroma development (Gerlis, 1956; Schwartz and Mitchell, 1962). Yet, it was not until the advent of immunohistological reagents in the 1980s which allowed the visualization of specific types of lymphocytes that the presence of B cells in atherosclerotic plaques and the adventitia of diseased blood vessels was clearly confirmed (Parums et al., 1990; Zhou and Hansson, 1999; Houtkamp et al., 2001). A wealth of active and passive immunization studies in mice and the identification of atheroprotective IgM antibodies, implicated B cells in attenuating atherosclerosis (Palinski et al., 1995; Ameli et al., 1996; Freigang et al., 1998; Nicoletti et al., 1998; Zhou et al., 2001; Binder et al., 2003, 2004; Faria-Neto et al., 2006). As new findings on the role of B cells in atherosclerosis have recently emerged, and in keeping with the theme of this series, this review will focus on the role of B cells in atherosclerosis.

## B Cells in Atherosclerosis

Two groups in 2002 directly tested the hypothesis that B cells modulate atherosclerosis. Caligiuri et al. reported that splenectomy of the atherogenic apolipoprotein-E knockout (*Apoe^−/−^*) mouse exacerbated atherosclerosis compared to the sham operated control mouse. Adoptive transfer of splenic B cells from atherosclerotic *Apoe^−/−^* mice not only rescued these mice from the atherogenic effects of splenectomy, but also reduced atherosclerosis to significantly less than that observed in the non-splenectomized controls. In addition, adoptive transfer of B cells, but not T cells, from atherosclerotic *Apoe^−/−^* mice to non-splenectomized, sham operated mice significantly attenuated atherosclerosis (Caligiuri et al., 2002). Consistent with these findings, Major et al. reported increased atherosclerosis in atherogenic LDL receptor knockout (*Ldlr^−/−^*) mice transplanted with bone marrow from B cell deficient (μ*MT*) mice compared to *Ldlr^−/−^* mice transplanted with bone marrow from C57BL/6 mice (Major et al., 2002). More recent studies confirmed a protective role for B cells in atherosclerosis. Lewis et al. demonstrated that *Ldlr^−/−^* mice unable to secrete IgM (*sIgM*) had accelerated atherosclerosis compared to control *Ldlr^−/−^* mice when fed a Western diet (Lewis et al., 2009). Doran et al. demonstrated marked attenuation of Western diet-induced atherosclerosis in B cell deficient μ*MT Apoe^−/−^* mice with the adoptive transfer of splenic B cells from *Apoe^−/−^* mice (Doran et al., 2012). Taken together, these studies indicate that B cells protect from Western diet-induced atherosclerosis.

**Figure 2:**
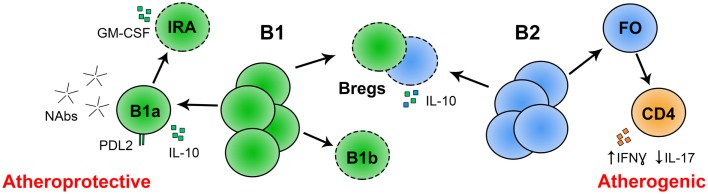
**Known and putative roles for B cell subsets in atherosclerosis**. Conventional, follicular B-2 B cells may promote atherosclerosis by skewing CD4 T cell differentiation to IFNγ producing Th1 cells and away from IL-17 producing Th17 T cells. The role of Bregs in atherosclerosis is not yet determined, but they may attenuate atherosclerosis by secretion of IL-10. Peritoneal B-1a B cells attenuate atherosclerosis through production of IgM, and potentially IL-10. PD-L2 is expressed on anti-PC B-1a B cells, potentially marking atheroprotective cells within this subset. The role of innate response activator B cells (IRA; derived from peritoneal B-1a B cells) in atherosclerosis is unknown but they produce GM-CSF, which may be linked to atherogenesis. The role of B-1b B cells in atherosclerosis is unknown. *(- - -) Role in atherosclerosis not yet reported.

In contrast, in 2010 two groups utilized an anti-CD20 monoclonal antibody to deplete B cells in *Apoe^−/−^* mice and found attenuation of Western diet-induced atherosclerosis (Ait-Oufella et al., 2010; Kyaw et al., 2010). Confirmation of an atherogenic role for B cells was provided by these same two groups in studies using atherosclerosis-prone mice null for B cell activation factor receptor (*Baffr^−/−^*) (Kyaw et al., 2012; Sage et al., 2012). *Baffr^−/−^* mice lack B-2 B cells that require BAFF for survival, such as follicular or marginal zone B cells (Mackay and Browning, 2002; Sasaki et al., 2004). *Baffr^−/−^ Apoe^−/−^* mice developed less severe atherosclerosis compared to control *Apoe^−/−^* mice when fed an atherogenic diet (Kyaw et al., 2012). Additionally, *Ldlr^−/−^* mice reconstituted with bone marrow from *Baffr^−/−^* mice had less Western diet-induced atherosclerosis compared to *Ldlr^−/−^* mice reconstituted with bone marrow from C57BL/6 mice (Sage et al., 2012). These studies suggest that B cells can aggravate atherosclerosis development. The apparent discrepancy in findings between studies suggesting an atheroprotective role for B cells and those suggesting an atherogenic role for B cells may be explained by unique roles for specific B cell subsets in regulating atherosclerosis. Indeed, anti-CD20 monoclonal antibody treatment and deletion at the *Baffr* locus predominantly depleted B-2 cells but not B-1a B cells (Mackay and Browning, 2002; Sasaki et al., 2004; Hamaguchi et al., 2005; Ait-Oufella et al., 2010; Kyaw et al., 2010, 2012; Sage et al., 2012). Below we briefly describe B cell subsets, followed by known and putative roles of these B cell subsets in atherosclerosis (Figure 2).

## B Cell Subsets

B cells can be divided into two developmentally distinct lineages, B-1 and B-2. These lineages arise in overlapping waves within a layered immune system where B-1 B cell development predominates in the fetus and B-2 B cell development in the adult. B-2 B cells include follicular B cells and marginal zone B cells; and B-1 B cells include B-1a B and B-1b B cells (Kantor and Herzenberg, 1993; Rothstein, 2002; Herzenberg and Tung, 2006; Baumgarth, 2011; Montecino-Rodriguez and Dorshkind, 2012). Common surface markers used to identify these B cell subsets are outlined in Table 1. Conventional follicular B-2 B cells undergo isotype switching and affinity maturation in the spleen and lymph nodes in response to T-dependent antigens to either become plasma cells that secrete large amounts of antibody, or memory B cells with the ability to produce specific antibodies upon re-exposure to the same antigen (Rajewsky, 1996; Tarlinton, 2006; Allen et al., 2007; Fairfax et al., 2008). Unlike conventional follicular B-2 B cells of the adaptive immune system, marginal zone B cells are considered part of the innate immune system. Marginal zone B cells reside in the spleen and are positioned to immediately respond to antigens in the blood that are filtering through the spleen (Martin and Kearney, 2002; Pillai et al., 2005).

**Table 1:** **B cell subset markers**.

Subset	Surface markers*	Citation
Follicular	CD19^+^ B220^+^ IgM^dull^ IgD^hi^ CD21^mid^ CD23^+^	Pillai and Cariappa (2009)
Marginal zone	CD19^+^ B220^+^ IgM^hi^ IgD^dull^ CD1d^hi^ CD21^hi^ CD23^−^	Pillai and Cariappa (2009)
B-1a	CD19^+^ B220^low/mid^ IgM^hi^ IgD^dull^ CD43^+^ CD11b^+^ CD5^+^	Hardy and Hayakawa (2001)
B-1b	CD19^+^ B220^low/mid^ IgM^hi^ IgD^dull^ CD43^+^ CD11b^+^ CD5^−^	Hardy and Hayakawa (2001)

*Surface markers define follicular and marginal zone B cells in the spleen, and B-1 cells in the peritoneal cavity.

Mature, adult B-1 B cells develop from fetal tissues including the liver and bone marrow, and less so from progenitors in the adult spleen and bone marrow (Montecino-Rodriguez et al., 2006; Esplin et al., 2009; Holodick et al., 2009; Barber et al., 2011; Ghosn et al., 2011; Montecino-Rodriguez and Dorshkind, 2012). Mature B-1 B cells are primarily found in serosal cavities and the spleen and have the capacity to self-renew. B-1 B cells are largely T cell-independent and produce the majority of IgM antibodies that recognize self and foreign antigens (Tumang et al., 2004; Hardy, 2006; Baumgarth, 2011). B-1a B cells spontaneously produce natural IgM antibodies, which constitutes most of the serum IgM at homeostasis (Forster et al., 1991; Mond et al., 1995a,b; Baumgarth et al., 1999; Ehrenstein and Notley, 2010). Additionally, their antibody repertoire is biased toward self-reactivity (Hardy et al., 1989; Mercolino et al., 1989; Pennell et al., 1989; Wang and Clarke, 2004; Rowley et al., 2007). B-1b B cells have a distinct role from B-1a B cells in that they can be induced to secrete antibodies by cross-linking the BCR on antigen specific cells. This response includes producing IgM or isotype switching to IgG3 or IgA, and may lead to unconventional memory formation (Hsu et al., 2006; Obukhanych and Nussenzweig, 2006; Alugupalli, 2008; Foote and Kearney, 2009; Haas, 2011).

Regulatory B cells (Bregs) are defined by their ability to inhibit autoimmune pathogenesis and restore tissue homeostasis mainly through production of IL-10 (Bouaziz et al., 2008; Lund and Randall, 2010; Mauri and Blair, 2010; Klinker and Lundy, 2012; Mauri and Bosma, 2012). Varying proportions of IL-10-producing B cells are found in several B cell subsets such as transitional 2-MZ precursor cells, MZ cells, and B-1a B cells, making it difficult to define a particular set of surface markers for Bregs. At present, the lack of clarity with respect to surface immunophenotype makes understanding Breg development challenging (Mauri and Bosma, 2012).

## B-1a B Cells in Atherosclerosis

Kyaw et al. recently confirmed the hypothesis that B-1a B cells can protect from atherosclerosis. Consistent with previous data, they demonstrated that splenectomized mice contain fewer B-1a B cells (Wardemann et al., 2002) and increased atherosclerosis (Caligiuri et al., 2002). Adoptive transfer of B-1a B cells attenuated splenectomy-aggravated atherosclerosis. B-1a B cells produce IgM antibodies that have long been implicated in atheroprotection (Binder et al., 2005). Indeed, adoptive transfer of B-1a B cells from *sIgM* mice did not protect from splenectomy-aggravated atherosclerosis (Kyaw et al., 2011).

The mechanisms whereby B-1a B cell-derived IgM can protect from atherosclerosis have been best characterized using the prototypic monoclonal IgM antibody, E06 (Chang et al., 1999, 2004; Hörkkö et al., 1999; Shaw et al., 2000; Binder et al., 2003; Chou et al., 2009). E06 is considered a natural antibody because it is structurally and functionally similar to the classic antibody T15, and is produced by B-1a B cells at homeostasis in germ-free mice (Shaw et al., 2000; Chou et al., 2009). It was cloned from spleens of Western diet fed *Apoe^−/−^* mice and is able to bind oxidized LDL (oxLDL) in serum and in atherosclerotic lesions (Palinski et al., 1996). E06 inhibits uptake of oxLDL by macrophages (Bird et al., 1999; Hörkkö et al., 1999), preventing foam cell formation, inhibiting release of pro-inflammatory cytokines in the atherosclerotic lesion. Additionally, E06 binds to epitopes on membrane phospholipids of pro-inflammatory apoptotic cells and mediates apoptotic cell clearance (Chen et al., 2009; Chou et al., 2009). Effective clearance of apoptotic cells and oxLDL neutralization reduces pro-inflammatory effects on other vessel wall cells (Chang et al., 1999, 2004; Huber et al., 2002; Binder et al., 2005; Tabas, 2010). Natural IgM antibodies, typified by E06, provide one mechanism whereby B-1a B cells can attenuate atherosclerotic plaque progression. This important class of antibodies and their role in innate immunity and atherosclerosis are reviewed in detail by Grönwall et al. (2012) in this series.

Characterization of the impact of splenectomy and B-1a B rescue on atherosclerotic plaques provided *in vivo* support for atheroprotective mechanisms originally described *in vitro* (Binder et al., 2005; Kyaw et al., 2011; Miller et al., 2011; Grönwall et al., 2012). Splenectomy led to reduced atherosclerotic lesion IgM content and increased the size of the necrotic core within the lesion (Kyaw et al., 2011). Large lesional necrotic cores typify advanced unstable plaques and are linked to failed apoptotic cell clearance (Seimon and Tabas, 2009; Tabas, 2010). Consistent with previous work demonstrating increased IgM in atherosclerotic lesions of *Rag^−/−^* mice after adoptive transfer of B-1 cells (Chou et al., 2009), adoptive transfer of B-1a B cells to splenectomized mice increased lesional IgM. The increased IgM was associated with a reduced necrotic core, an effect that was lost when the transferred B-1a B cells came from sIgM null mice (Kyaw et al., 2011). These data suggest that the increased necrotic core could be due to failed IgM-mediated clearance of apoptotic foam cells. Indeed, adoptive transfer of B-1a B cells reduced splenectomy-induced lesional apoptosis. However, it is not clear that this effect was dependent on IgM, as there was a trend toward an increase in apoptotic cells in lesions from mice receiving B-1a B cells null for sIgM compared to wildtype, but it was not statistically significant (Kyaw et al., 2011). Consistent with this observation, *sIgM* *Ldlr^−/−^* mice had only a non-significant trend toward an increase in lesion apoptotic cell content compared to control *Ldlr^−/−^* mice (Lewis et al., 2009). Additional studies will be needed to fully address the role of B-1a B cell-derived IgM in apoptotic clearance *in vivo*. Moreover, IgM-dependent effects of B-1a B cells may need to be tested in a model without splenectomy as B-1a B cells may need the spleen for full IgM production. B-1a B cells in the peritoneal cavity spontaneously secrete low amounts of IgM, but in the spleen, they are “super-secretors” (Holodick et al., 2010), suggesting that the spleen may be important for B-1a B cells to produce large amounts of IgM antibodies against modified lipids that are protective against atherogenesis. In addition, these results raise the possibility that B-1a B cells may also regulate apoptotic cell clearance by mechanisms independent of IgM. B-1a B cells are known to produce cytokines involved in atheroprotection (O’Garra et al., 1992) and future studies are needed to determine if production of cytokines might be another mechanism whereby B-1a B cells protect from atherosclerosis (Figure 2).

Many other questions about the role of B-1a B cells in atherosclerosis remain. IgM that recognizes apoptotic cells and oxLDL are the most well studied natural antibodies, but are there other protective antibodies that regulate atherosclerosis? Are there harmful autoreactive antibodies that have pro-atherogenic effects? Are there specific subtypes of B-1a B cells that have atheroprotective functions? Programmed death-1 ligand 2 (PD-L2), a ligand for PD-1, is expressed on 50–70% of B-1a B cells. The PD-L2 positive B-1a B population has a biased immunoglobulin repertoire for self-reactivity, can present antigen more potently, induce Th17 formation, and can switch isotype more readily than PD-L2 negative B-1a B cells (Figure 2) (Zhong et al., 2007a,b; Zhong and Rothstein, 2011; Wang and Rothstein, 2012). Might PD-L2 mark an atheroprotective population within the B-1a B cell subset? Additionally, do B-1a B cells function locally in the aorta? Are there functionally relevant numbers of B-1a B cells in the adventitia or surrounding peri-aortic adipose tissue? Do they have different roles at homeostasis and early disease compared to late disease? B-1b B cells also produce IgM and can undergo clonal expansion in response to foreign antigen (Viau and Zouali, 2005; Hardy, 2006; Baumgarth, 2011). Yet, do B-1b B cells act in atherosclerosis (Figure 2)?

## B-2 B Cells in Atherosclerosis

B cell depletion studies have suggested that B-2 B cells are an atherogenic B cell subset. In support of this finding, adoptive transfer of 5 × 10^6^ splenic B-2 cells from a C57BL/6 background, aggravated atherosclerosis in B cell deficient μ*MT* *Apoe^−/−^* mice fed 6 weeks of Western diet (Kyaw et al., 2010). The mechanisms by which B-2 B cells can aggravate atherosclerosis are incompletely understood. Anti-CD20 treatment was associated with an increase in the percentage of IL-17+ T cells (Th17 cells), and IL-17A neutralization abrogated anti-CD20 attenuation of atherosclerosis (Ait-Oufella et al., 2010). These results imply that IL-17 may mediate B-2 cell aggravation of atherosclerosis (Figure 2). However, the role for IL-17 in atherosclerosis remains controversial (Erbel et al., 2009; Taleb et al., 2009; van Es et al., 2009; Gao et al., 2010; Smith et al., 2010; Butcher et al., 2012; Danzaki et al., 2012). In addition to increasing Th17 cells, anti-CD20 treatment was also associated with a decrease in CD4 T cell secretion of the Th1 cytokine IFNγ, and reduced proliferation and activation of splenic CD4 T cells (Ait-Oufella et al., 2010; Sage et al., 2012). Several pro-atherogenic roles for Th1 cells have been identified and reviewed (Zhou, 2003; Taleb et al., 2010; Dumitriu and Kaski, 2011; Hansson and Hermansson, 2011; Lahoute et al., 2011; Weber and Noels, 2011; Campbell et al., 2012). Depletion of B-2 B cells was also associated with decreased T cells in the atherosclerotic plaque (Ait-Oufella et al., 2010; Kyaw et al., 2012; Sage et al., 2012), suggesting that B-2 B cells may aggravate atherosclerosis by regulating T cells in the aorta as well as the spleen.

B-2 B cells may also aggravate atherosclerosis by producing pathogenic antibodies. B-2 B cell depletion was associated with a reduction in total serum IgG including IgG1, IgG2a, IgG2c, as well as IgG1 and IgG2a in the atherosclerotic plaque. Furthermore, B-2 cell depletion resulted in a reduction in serum IgG against modified lipids, oxLDL and malondialdehyde LDL (MDA-LDL). Consistent with the predominant depletion of B-2 cells and not B-1a B cells, there were only modest decreases in total IgM and IgM against MDA-LDL and oxLDL (Ait-Oufella et al., 2010; Kyaw et al., 2010, 2012; Sage et al., 2012). Interestingly, univariate analysis revealed that serum levels of IgG and IgM to oxLDL have divergent associations with coronary artery disease in humans. IgM to OxLDL was inversely associated with coronary artery disease while IgG was positively associated (Tsimikas et al., 2007). Mechanisms whereby adaptive immunoglobulins might regulate plaque development are poorly understood. Downstream of antibody production, B cells may indirectly regulate atherosclerosis in an antigen-independent manner when IgG immune complexes bind to Fc gamma receptors (FcγR). Activating FcγRs have been implicated as being pro-atherogenic (Hernandez-Vargas et al., 2006) and inhibitory FcγRIIb anti-atherogenic (Kelly et al., 2010; Mendez-Fernandez et al., 2011). Additionally, IgE and its Fc receptor present on mast cells, FcεR1α, are pro-atherogenic (Wang et al., 2011).

B-2 B cells may also promote atherosclerosis by altering other inflammatory mediators in the aorta. The loss of BAFFR in *Apoe^−/−^* mice resulted in decreased immunostaining of VCAM1, CD11c, CD83 and PCNA, and reduced gene expression of the inflammatory markers TNFα, IL1β, and MCP1 in the atherosclerotic lesion (Kyaw et al., 2012). Presumably, these changes are due to loss of B-2 cells. Although, other BAFFR-dependent mechanisms may regulate these changes as BAFFR may also be expressed on T cells (Ye et al., 2004). Many questions about the role of B-2 cells in atherosclerosis remain. What activates the adaptive immune system in atherosclerosis? More specifically, what activates B cells? Do conventional B-2 cells respond to activation and produce antibodies that directly regulate atherosclerosis in a protective or pathogenic way? Might atherosclerosis be an allergic disease (Binder and Witztum, 2011)?

## Regulatory B Cells in Atherosclerosis

As regulatory B cells produce IL-10 (Madan et al., 2009; Saraiva and O’Garra, 2010; Mauri and Bosma, 2012), the likely hypothesis is that they are atheroprotective (Figure 2). Mice null for IL-10 develop significantly more atherosclerosis than controls (Mallat et al., 1999a; Pinderski Oslund et al., 1999). IL-10 has been reported to protect from atherosclerosis by inhibiting production of pro-inflammatory mediators and apoptosis, and modulating lipid metabolism (Mallat et al., 1999a; Von Der Thüsen et al., 2001; Binder et al., 2003; Caligiuri et al., 2003). Bregs also express fas ligand and tumor necrosis factor-related apoptosis-inducing ligand (Von Der Thüsen et al., 2001; Mauri and Bosma, 2012). These molecules induce apoptosis in target cells through cell–cell interactions, which may be an important mechanism for suppressing pro-inflammatory immune responses in atherosclerotic lesions. Surface markers that define Bregs are poorly understood, making their function in atherosclerosis hard to study. Indeed, a direct role for regulatory B cells in atherosclerosis has not yet been reported.

## Context and Timing of B Cell Function in Atherosclerosis

The role of B cells in regulating atherosclerosis is likely more complex than just simple subset distinction. It is likely that exogenous factors impact on B cell subsets to alter their biology. For example, a newly described population of effector B cells, termed “innate response activator” (IRA) B cells is derived from peritoneal B-1a B cells. Adoptive transfer of B-1a B cells from CD45.2 mice to the peritoneal cavity of LPS-treated CD45.1 mice demonstrated that B-1a B cells can convert to granulocyte-macrophage colony-stimulating factor (GM-CSF) producing IRA B cells in the spleen (Rauch et al., 2012). GM-CSF converts Ly6C^lo^ monocytes to pathogenic Ly6C^hi^ monocytes in the spleen, which can then traffic to atherosclerotic lesions and become atherogenic foam cells, exacerbating atherosclerosis (Zhou, 2003). It is important to note that these cells change their surface marker expression from CD19^+^B220^lo^CD11b^±^CD5^+^CD43^+^ in the peritoneal cavity to CD19^+^B220^hi^CD11b^−^CD5^+^CD43^+^ in the spleen (Rauch et al., 2012), demonstrating environment-dependent B-1a B cell plasticity with respect to surface marker expression (Tumang et al., 2004; Steinberg and Witztum, 2010). A direct role for IRA B cells in atherosclerosis has yet to be determined (Figure 2).

The importance of context is also suggested by data demonstrating that adoptive transfer of 30 × 10^6^ splenic B-2 cells from Apoe^−/−^ mice attenuated atherosclerosis in B cell deficient μ*MT* *Apoe^−/−^* mice fed 16 weeks of Western diet. Transfer of 60 × 10^6^ *Apoe^−/−^* splenic B-2 cells had an even greater attenuation of atherosclerosis in μ*MT* *Apoe^−/−^* mice (Doran et al., 2012). These findings are in apparent contrast to the adoptive transfer studies of Kyaw et al. (transferred 5 × 10^6^ splenic B-2 cells from a C57BL/6 mice to μ*MT* *Apoe^−/−^* mice) suggesting that the number of B cells transferred or differences in genotype (*Apoe^−/−^* vs. C57BL/6) or phenotype (hyperlipemic or normolipemic) of the donor mice may be important (Kyaw et al., 2010; Lipinski et al., 2011). Indeed, Caligiuri et al. reported a greater attenuation of atherosclerosis with splenocytes transferred from older atherosclerotic mice compared to C57BL/6J mice or young *Apoe^−/−^* mice (Caligiuri et al., 2002).

**Figure 3:**
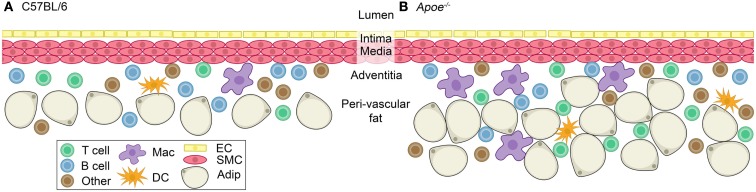
**Immune cells at homeostasis in the adventitia of C57BL/6 and *Apoe^−/−^* mice**. A healthy blood vessel is composed of an endothelial layer, an intima in between the endothelial layer and smooth muscle cell layer, and a smooth muscle cell layer (media) surrounded by the adventitia and peri-vascular fat. **(A)** The adventitia of a C57BL/6 mouse contains T cells, B cells, macrophages, dendritic cells, and others (neutrophils, natural killer cells, and natural killer T cells; Galkina et al., 2006; Jongstra-Bilen et al., 2006). **(B)** *Apoe^−/−^* mice have an increase in the number of T cells and macrophages, with a lesser increase in dendritic cells and other cells (neutrophils, natural killer cells, and natural killer T cells) compared to C57BL/6 (Galkina et al., 2006). Abundant adventitial B cells persist in the *Apoe^−/−^* mouse (Galkina et al., 2006; Doran et al., 2012). They also have an expanded peri-vascular fat pad compared to **(A)**. Abbreviation: Mac, macrophage; DC, dendritic cell; EC, endothelial cell; SMC, smooth muscle cell; Adip, adipocyte.

In addition, evidence suggests that B cells may have specific functions in their local environment in the aorta. Doran et al. utilized an *Apoe^−/−^* mouse null for Id3 to explore the importance of vessel wall B cells in atherosclerosis. Id3, a helix loop helix transcription factor, and its partner proteins are important for B cell development and function (Pan et al., 1999; Engel and Murre, 2001; Murre, 2005). *Apoe^−/−^* and *Apoe^−/−^* *Id3*^−*/*−^ mice contained an equal number of splenic and circulating B cells, consistent with prior reports (Pan et al., 1999). However, significantly fewer B cells, and a marked increase in atherosclerosis, were detected in the aortas of *Apoe^−/−^* *Id3*^−*/*−^ mice. Similar results have subsequently been reported in *Ldlr^−/−^* mice (Lipinski et al., 2012). Moreover, in contrast to splenic B cells from wildtype *Apoe^−/−^* mice, adoptively transferred splenic B cells from *Apoe^−/−^* *Id3*^−*/*−^ mice did not home to the aorta of μ*MT Apoe^−/−^* mice, and subsequent analysis revealed no difference in atherosclerosis. Interestingly, radiolabeled splenic B cells from *Apoe^−/−^* mice adoptively transferred to μ*MT Apoe^−/−^* mice predominantly homed to specific sites within the aorta suggesting regional preferences for homeostatic B cell trafficking to the vessel wall. B cells appear to home and reside in regions prone to atherosclerotic disease (Doran et al., 2012; Lipinski et al., 2012), and loss of B cells in these locations is associated with an increase in atherosclerosis development. Notably, the above studies evaluating immune cell composition of the aorta by flow cytometry and B cell homing by imaging were performed in mice prior to the development of atherosclerosis, suggesting that resident immune cells in the aorta at baseline are important for the response to atherogenic stimuli (Figure 3). In this context, B cells were linked to atheroprotection. These B cells may represent the innate arm of B cell-mediated responses to atherogenic stimuli.

Innate immunity plays a major role in initial defense against disease by using natural receptors that discriminate “self” vs. “neo-self” epitopes. In the context of atherosclerosis, self epitopes, such as native LDL can become modified after oxidation to become neo-self epitopes in the form of MDA-LDL and oxLDL. Oxidation-specific epitopes are a class of “danger signals,” and are recognized by pattern recognition receptors in innate immunity, which include scavenger receptors, innate effector proteins, and natural IgM antibodies (Binder et al., 2005; Miller et al., 2011). In addition to oxidized lipids, lipid-laden macrophages that undergo apoptosis expose the phosphorylcholine (PC) group, a neo-self epitope, on oxidized phospholipids (oxPL) in their plasma membrane (Miller et al., 2011). One group of antibodies that recognizes the PC group on oxPLs and apoptotic cells are referred to as T15 antibodies, which include E06, and are present in atherosclerotic plaques (Shaw et al., 2000; Chou et al., 2009). A turning point in the progression of atherosclerosis is the failure to resolve inflammation, which usually involves the suppression of cell infiltration, effective clearance of apoptotic cells, and promotion of cell efflux from the arterial wall. However, when there are defects in these mechanisms, or when these mechanisms are overwhelmed, atherosclerotic plaques progress to dangerous plaques capable of rupture (Tabas, 2010). Lipid antigen and apoptotic cells may overwhelm innate immunity and stimulate the adaptive immune system, resulting in loss of immune tolerance, and a stimulation of increased autoantibodies (Miller et al., 2011; Weih et al., 2012).

B cells may have different functions at later stages of atherosclerosis. At homeostasis, dendritic cells, macrophages, T cells and B cells are present in the adventitia and peri-aortic adipose tissue in wildtype and hyperlipidemic mice without atherosclerosis (Figure 3) (Galkina et al., 2006; Doran et al., 2012), indicating that the aortic adventitia and surrounding peri-vascular fat is a homeostatic niche for specific leukocytes including B cells. Most of these B cells at homeostasis are follicular B cells based on their surface marker expression (Table 1) with 1–2% B-1 B cells also being present (our unpublished observations). After 20 weeks of Western diet-induced atherosclerosis in *Apoe^−/−^* mice, the total number of macrophages, T cells, and dendritic cells, but not B cells, increase significantly (Galkina et al., 2006). This was associated with the formation of ATLOs (Figure 1) (Libby, 2012). Without Western diet, ATLOs and lesions form together at >52 weeks of age. These ATLOs are found mostly in the abdominal aorta and less so in the thoracic aorta, consistent with the thoracic aorta being devoid of lesions at early time points. In advanced atherosclerosis, ATLOs contain B cell follicles with germinal centers and follicular dendritic cell networks. Germinal centers show signs of activated B cells because they contain proliferating B cells surrounded by follicular mantle cells. Plasma cells are also present (Grabner et al., 2009). These observations suggest that at this advanced stage of disease, innate protection may be overwhelmed and adaptive responses with loss of tolerance may predominate. The fact that ATLOs are found adjacent to advanced plaques only in aged mice, has led to the notion that B cells in ATLOs are reactive and atherogenic (Weih et al., 2012). Taken together, these studies raise potentially important distinctions between homeostasis, where B cells may be protective (Doran et al., 2012), and advanced disease, where lipid antigen and apoptotic cells may overwhelm protective cells.

## B Cells in Human Atherosclerosis

How findings in murine models will apply to understanding human atherosclerotic disease pathogenesis or how this may impact on therapy remains unknown. The advantages of mouse models of atherosclerosis, such as vast genetic information, affordability, feasible genetic manipulation, and pharmaceutical testing, makes it a favorable tool for understanding disease pathology. However, the rate of development, location, and manifestations of atherosclerotic lesions studied in mice may differ from clinically significant lesions in humans (Schwartz et al., 2007; Zadelaar et al., 2007; Pendse et al., 2009; Bentzon and Falk, 2010; Getz and Reardon, 2012). In addition, surface markers that identify B cell subsets differ between mice and humans. While CD5 is a marker of B-1a cells in mice, it does not reliably discriminate between B-1 and B-2 cells in humans (Freedman et al., 1989; Sims et al., 2005; Lee et al., 2009; Griffin et al., 2011; Kaminski et al., 2012). A major step toward translating questions about the role of B cell subsets in the context of human atherosclerosis came with the elegant study by Griffin et al. that identified a circulating human B cell subset with functional properties similar to those associated with murine B-1 cells. Circulating B cells had been reported to produce IgM antibodies against modified lipids (Chou et al., 2009), but it was unknown which B cell fraction was responsible. The investigators tested sort-purified B cell fractions for four functions that typify murine B-1 cells: spontaneous IgM secretion, contain PC-binding antigen receptors, efficient T cell stimulation, and tonic intracellular signaling. The human B cell fraction that met these criteria was CD20^+^CD27^+^CD43^+^ (Griffin et al., 2011). Follow on studies identified CD11b^+^CD20^+^CD27^+^CD43^+^ B cells termed orchestrator B-1 cells that are increased in SLE patients, spontaneously produce IL-10, and suppress T cell activation. IL-10 has been shown to be associated with protective functions in human atherosclerosis, consistent with mouse data (Uyemura et al., 1996; Mallat et al., 1999b; Smith et al., 2001; Heeschen et al., 2003; Fichtlscherer et al., 2004). CD11b^−^ B-1 cells primarily secrete IgM antibody are termed secretor B-1 cells (Griffin and Rothstein, 2011, 2012). Notably, the percentage of circulating human CD27^+^ B cells belonging to the B-1 subset declines with age in a pattern strikingly similar to the inverse of the age-related increase in atherosclerosis prevalence. Moreover, published studies provide evidence that circulating levels of IgM that bind modified lipids, which are inversely associated with coronary artery disease, decline with age (Tsimikas et al., 2007). Taken together, it is very intriguing to hypothesize that the circulating human subset identified by Griffin et al. may play an atheroprotective role in humans.

How to test the hypothesis that human B-1 cells are atheroprotective is the challenge. While the promise of translating important findings in murine models of atherosclerosis to humans concludes the discussion section of hundreds of important scientific papers, including those reporting on the role of B cells in atherosclerosis, the reality is that this translation is rarely realized at this juncture. This is not for want of such translation, but rather due to significant practical limitations. The expense and duration of clinical end-point trials is one such barrier. As such, identification and utilization of surrogate markers of atherosclerosis-based clinical events is essential (Choi et al., 2008; Fraley et al., 2009; Weismann et al., 2011; Purushothaman et al., 2012). Our group is currently utilizing coronary artery intravascular ultrasound (IVUS) in humans to quantitate the amount of coronary artery atherosclerotic plaque and determine if associations exist between plaque burden and the percentage of circulating B cells that belong to the CD20^+^CD27^+^CD43^+^ subset. Given the age-related decline in circulating CD20^+^CD27^+^CD43^+^ cells and the multiple covariates linked to atherosclerosis that exist in a human population, this study will need a large number of subjects and a large amount of investigator perseverance.

## Concluding Remarks

While a myriad of questions remain unanswered, our understanding of the role played by B cells in murine models of atherosclerosis has made significant leaps in the last decade. If we are to translate these important murine findings to humans and unravel the role of B cells in human atherosclerosis we need to start testing for novel B cell phenotypes associated with clinical or subclinical atherosclerotic cardiovascular disease. Hopefully, these associations will lead to new mechanistic hypotheses that can be tested in prospective, controlled clinical studies.

## Conflict of Interest Statement

The authors declare that the research was conducted in the absence of any commercial or financial relationships that could be construed as a potential conflict of interest.
